# Are We Making Personalized Cancer Care Less Personalized?

**DOI:** 10.3389/fonc.2016.00220

**Published:** 2016-10-20

**Authors:** Steven Sorscher

**Affiliations:** ^1^Wake Forest Medical School, Winston Salem, NC, USA

**Keywords:** personalized medicine, cancer, therapies, predictive modeling, decision making

To most physicians and their patients, personalized cancer care typically means that after identifying specific so-called “actionable” molecular changes characterizing a patient’s cancer, a highly effective therapy that specifically targets the expressed product of that abnormality is available. This is now the case for the three most common US causes of cancer death (1–3). Personalized cancer care also means studying the molecular nature of the tumor to estimate the benefits of adjuvant systemic therapy. For example, after surgery for most breast cancers, the National Comprehensive Cancer Network now endorses quantitative mRNA expression profiling in order to estimate the absolute benefit of adjuvant hormonal therapy or adjuvant chemotherapy followed by hormonal therapy for that patient (4).

William Osler said, “It is much more important to know what sort of person has a disease than what sort of disease a person has.” The other aspect of personalized cancer care is understanding the patient who harbors the cancer. For example, geriatric assessment tools (GATs) can be used to help more precisely estimate the risks and benefits of particular treatments in older patients (5). Similarly, assessing genomic polymorphisms can be used to estimate certain risks and benefits of specific therapies in particular patients (6).

There is a danger that results from relying too heavily on these tools to direct patients in decision making. For example, we have all cared for patients who choose against a highly effective targeted agent and other patients who wish to pursue therapies even when the tools mentioned above offer a far more dysphemistic prediction of efficacy and risk. This is because what informs patient decisions is often far more intangible and relates to the fundamental values, desires, and the nature of a particular patient.

Futurists predict that soon computer applications will be capable of making nearly all medicine-related treatment decisions. However, I doubt there will ever be a laboratory or clinical tool that can estimate the love for family that motivates a patient to pursue options, against all odds, in order to be there for a marriage, graduation, or birth of a grandchild. We will never “personalize” cancer care to help patients quantify the beauty of a poem or song and appreciate the wonder of an intelligent conversation or the joy of sharing laughter with a friend. The promise of truly personalized care rests on taking the time to listen to the value patients place on these intangibles as they consider their options in the context of our increased and remarkable ability to more precisely analyze the tumor and other more concrete patient-related factors.

## Author Contributions

The author confirms being the sole contributor of this work and approved it for publication.

## Conflict of Interest Statement

There are no relevant conflicts related to this publication.
